# The probiotic *Lacticaseibacillus rhamnosus* HN001 influences the architecture and gene expression of small intestine tissue in a piglet model

**DOI:** 10.1017/S0007114523002830

**Published:** 2024-04-28

**Authors:** Carlos A. Montoya, Wayne Young, Leigh Ryan, Kelly Dunstan, Jason Peters, Hilary Dewhurst, James Dekker, Neill Haggarty, Ryan N. Dilger, Nicole C. Roy

**Affiliations:** 1 Smart Foods & Bioproducts, AgResearch, Te Ohu Rangahau Kai Facility, Palmerston North, New Zealand; 2 Riddet Institute, Massey University, Te Ohu Rangahau Kai Facility, Palmerston North 4474, New Zealand; 3 High-Value Nutrition National Science Challenge, Auckland, New Zealand; 4 Fonterra Research and Development Centre, Dairy Farm Rd, Palmerston North, New Zealand; 5 Department of Animal Sciences, University of Illinois, Urbana, IL, USA; 6 Department of Human Nutrition, University of Otago, Dunedin, New Zealand

**Keywords:** *Lacticaseibacillus rhamnosus* HN001, Piglet, Tissue architecture, Gene expression, Jejunum, Ileum

## Abstract

This study investigated the effects of *Lacticaseibacillus rhamnosus* HN001 supplementation on the architecture and gene expression in small intestinal tissues of piglets used as an animal model for infant humans. Twenty-four 10-d-old entire male piglets (4·3 (sd 0·59) kg body weight) were fed an infant formula (IF) (control) or IF supplemented with 1·3 × 10^5^ (low dose) or 7·9 × 10^6^ (high dose) colony-forming units HN001 per ml of reconstituted formula (*n* 8 piglets/treatment). After 24 d, piglets were euthanised. Samples were collected to analyse the histology and gene expression (RNAseq and qPCR) in the jejunal and ileal tissues, blood cytokine concentrations, and blood and faecal calprotectin concentrations. HN001 consumption altered (false discovery rate < 0·05) gene expression (RNAseq) in jejunal tissues but not in ileal tissues. The number of ileal goblet cells and crypt surface area increased quadratically (*P* < 0·05) as dietary HN001 levels increased, but no increase was observed in the jejunal tissues. Similarly, blood plasma concentrations of IL-10 and calprotectin increased linearly (*P* < 0·05) as dietary HN001 levels increased. In conclusion, supplementation of IF with HN001 affected the architecture and gene expression of small intestine tissue, blood cytokine concentration and frequencies, and blood calprotectin concentrations, indicating that HN001 modulated small intestinal tissue maturation and immunity in the piglet model.

Probiotic bacteria are increasingly being added to infant formulas (IF) to enhance gastrointestinal tract (GIT) health by modulating the gut microbiota composition or the mucosal immune system^(1–5)^. For instance, infants fed a *Lactobacillus reuteri*-supplemented IF for 12 weeks (including follow-up) had a 4-fold lower incidence of diarrhoea than those fed un-supplemented IF^(6)^. Furthermore, children orally dosed with a combination of *L. rhamnosus* strains 573L/1, 573L/2 and 573L/3, at 10^10^ colony-forming units (CFU) twice a day for five consecutive days, showed reduced duration of acute rotavirus diarrhoea by 39 h and reduced the need for intravenous rehydration by 23 h^(1)^.

It has been previously shown that the intake of *L. rhamnosus* HN001 from birth to 2 years of age reduced the relative risk of developing eczema and rhinoconjunctivitis^(7)^. In addition, other preclinical and clinical studies have shown that HN001 also had beneficial effects on GIT health and immunity^(8–10)^. For instance, HN001 increased blood and peritoneal leucocyte phagocytic capacity in mice^(9)^, improved GIT barrier function, decreased terminal ileum inflammation and modified intestinal immune responses in neonatal mice and preterm piglets^(9,11)^. Furthermore, Tannock *et al*.^(12)^ found that HN001 influenced the expression of different genes related to apoptosis (*sgk1*, *angptl4* and *hspa1b*) in the small intestine of both infant and adult mice. However, these studies have been mainly conducted in adult human or animal models, such that it remains unclear whether the benefits of HN001 on GIT health can be replicated in human infants, and if so, what mechanisms might be involved.

Therefore, it was hypothesised that HN001 could influence the architecture and gene expression of the small intestinal tissue in piglets as a model of human infants. This study aimed to investigate the effect of supplementing IF with two levels of HN001, a lower dose typical of commercial IF and a higher dose more typical of probiotic supplements. The impacts of dietary HN001 on the histology and gene expression of jejunal and ileal tissues of piglets were then assessed. Only the small intestine was investigated in this study as previous work showed that HN001 abundance relative to the total microbiota, as measured by 16S rRNA, reached from 3 % in jejunal to 30 % in ileal digesta of adult mice given HN001 in drinking water, whereas it was negligible in caecal and proximal colonic digesta. Piglets were used as an animal model for the infant human as they share greater similarities with neonatal humans in terms of the anatomical and physiological development of the GIT^(13,14)^.

## Materials and methods

An experimental protocol was prepared and approved by the research team prior to starting the study. Ethics approval for the study was obtained from the Animal Ethics Committee, AgResearch Limited, Palmerston North, New Zealand (approval number 13982).

### Animals and dietary treatment

Twenty-four entire male piglets, 10 d of age (Hampshire × (Landrace × arge white), 4·3 (sd 0·59) kg body weight (BW)) were purchased from a commercial farm (Aorere Farms). Piglets were housed in pairs during the first 2 d of arrival (adaptation period) in cages (1·0 m × 0·5 m) with an automated nutritional dispensing system as described earlier^(15)^. Piglets received a reconstituted nutritional base IF formulated by Fonterra Ltd during the adaptation period. The room temperature was maintained initially at 25°C and decreased 1°C per week thereafter.

Probiotic *Lacticaseibacillus rhamnosus* HN001 (formerly known as *Lactobacillus rhamnosus* HN001) (also known as LactoB HN001™) was supplied by Fonterra Ltd.

### Experimental design

On the third day after arrival, piglets (*n* 24) were housed individually and randomly assigned to each cage. Each piglet was then randomly assigned to one of three groups: IF control (piglets given IF without added HN001); HN001 low dose (IF with HN001 added at 1·3 × 10^5^ CFU/ml) or HN001 high dose (IF with HN001 added at 7·9 × 10^6^ CFU/ml) (*n* 8 piglets/treatment). Some of the researchers and all technical support staff were aware of the diet allocation to each piglet. During the study, clean toys were provided to the piglets. Every day, all piglets were together for 1 h to provide social contact while cages and food reservoirs were cleaned. Piglets were monitored at least twice a day for general health, alertness, dietary intake, signs of dehydration and scouring using a scoring system to determine whether piglets should remain in the study. BW was also monitored, with BW loss > 10 % over 7 d used as the cut-off to exclude piglets from the study.

Piglets received reconstituted IF treatments every 2 h via an automated system for 21 d. Food intake was recorded daily, and BW was recorded weekly. On day 20, fresh faeces were collected and stored at –80°C until analysis. On the last experimental day (i.e. day 21), piglets received at least three meals (two hourly intervals) before they were anaesthetised with 40 µl/kg BW of anaesthetic cocktail (Zoletil 100 (50 mg/ml), Ketamine (50 mg/ml) and Xylazine (50 mg/ml)). Blood samples were collected via cardiac puncture in EDTA-coated tubes, and piglets euthanised by a cardiac injection of sodium pentobarbitone (0·4 ml/kg BW of Pentobarb 300). Piglets were euthanised over two consecutive days, with order of euthanasia randomly allocated to each piglet to ensure that the same number of piglets per treatment was killed each day.

Blood samples were centrifuged at 2000 ***g*** for 10 min at room temperature. Plasma samples were then collected and snap-frozen in liquid N_2_ prior to being stored at –80°C. Finally, the abdominal cavity was opened, and the small intestine dissected out and uncoiled to collect jejunal (mid-small intestine) and ileal (20 cm prior to the ileocecal junction) tissues for both histology and gene expression analyses (as described below).

### Histology

Tissues collected for histological assessment were washed in ice-cold saline, submerged in 10 % formalin and stored at room temperature. The tissues were excised, dehydrated and embedded in paraffin wax. Each tissue section was cut in serial sections (5 µm), stained with haematoxylin and eosin, and periodic acid-Schiff-Alcian blue. Fifteen well-oriented villi and associated crypts per section were selected and measured using light microscopy at 40× magnification. The villus:crypt ratio was calculated by dividing villus height by its associated crypt depth. The total numbers of goblet cells in the villi of the jejunum and the crypts of the ileum were counted.

### Gene expression

Jejunal and ileal tissues collected for gene expression analysis were washed in ice-cold saline and snap-frozen in liquid N_2_ prior to being stored at –80°C until analysis. RNA was extracted from the tissues using Qiagen AllPrep kits (Bio-Strategy Limited) and reverse transcribed to cDNA using Applied Biosystems High-Capacity cDNA Reverse Transcription Kit (Thermo Fisher Scientific) for claudin 1 and 2, occludin, tight-junction protein 1 and 2, 14-3-3 protein zeta/delta (YWHAZ), β-2 microglobulin (β2M) and succinate dehydrogenase complex, subunit A (SDHA). Each sample was run in triplicate. Reference genes (YWHAZ, β2M and SDHA) were statistically analysed at each small intestinal location prior to data normalisation. β2M and SDHA gene expression was influenced by treatment group and/or the IF consumed on the sampling day. Thus, tight junction gene expression was normalised only using YWHAZ.

Gene expression profiles of jejunal and ileal tissue samples were also analysed by RNAseq. Total RNA was extracted using RNeasy Mini Kits (Qiagen). RNA quality was assessed using an Agilent 2100 Bioanalyser Instrument (Agilent), with samples with an RNA integrity threshold > 6·5 submitted for sequencing.

According to the manufacturer’s guidelines, strand-specific cDNA libraries were prepared using NEBNext® Ultra Directional RNA Library Prep Kit for Illumina® (Illumina).

Libraries were size selected for 250–300 bp fragments and sequenced using the Novaseq 6000 platform (Illumina) to produce 150 bp paired-end sequences. Reads were quality trimmed using Trimmomatic 0·36^(16)^. Read pairs that passed quality trimming were mapped against the genome (Scrofa 11·1 release 96) using STAR^(17)^. Uniquely mapped read pairs were summed for each gene and analysed using the EdgeR package^(18)^ within R statistical software. The Benjamini–Hochberg method was used for false discovery rate correction, as implemented in the lrt function from EdgeR. The resulting counts were analysed using a likelihood ratio generalised linear model, with genes that had > 1·5-fold difference (i.e. log fold change > |0·58|) with a false discovery rate < 0·05 considered differentially expressed. In addition, the enrichment analyses of genes annotated to Gene Ontology Biological Processes and Kyoto Encyclopedia of Genes and Genomes pathways within the list of differentially expressed genes were carried out using the ClueGO app^(19)^ for Cytoscape.

Changes in gene expression at the pathway level were also assessed using gene set enrichment analysis (GSEA) with the mroast function from limma^(20)^ and Kyoto Encyclopedia of Genes and Genomes pathways^(21,22)^ used as gene sets. GSEA involves the analysis of the aggregated expression of genes that share common biological functions, such as those within canonical pathways, rather than individual genes. Thus, GSEA methods can be useful to detect more subtle changes distributed across an entire network of genes that may be undetected at the level of individual genes^(23)^.

### Calprotectin, cortisol and α1-antitrypsin inhibitor promoter concentrations

The concentrations of calprotectin, cortisol and *α*1-antitrypsin inhibitor promoter in both faeces and blood plasma were determined using commercial ELISA kits (MyBioSource). Faeces (10 mg) were thoroughly mixed with 100 µl PBS, centrifuged (2000 ***g*** 20 min at 4°C) and the supernatant collected. The standards and samples were then analysed in duplicate as per the manufacturer’s instructions.

### Blood plasma cytokine concentrations

The Porcine Chemokine/Cytokine Panel 9plex from Affymetrix (Thermo Fisher Scientific) was used to measure different cytokines present in blood plasma (interferon (IFN)-*α*, IFN-*γ*, IL-10, IL-12p40, IL-4, IL-6, IL-8 and TNF-*α*). The standards and samples were analysed in duplicates as per the manufacturer’s instructions using a Bio-Rad FACSVerse flow cytometer.

### Statistical analysis

As this study was the first to investigate the effect of HN001 on the small intestine of piglets, previous specific gene expression data from the small intestine of mice fed HN001 were used to determine the sample size^(12)^. Based on the jejunal and ileal expression of the genes sgk1 and angptl4, it was calculated that a power > 0·8 could be reached with 7–9 animals. Thus, eight piglets per treatment were used.

Statistical analyses were performed using SAS for response variables (SAS/STAT version 9·4; SAS Institute Inc.) other than gene expression profiles (see the “gene expression” section above). Using the Proc Mixed of SAS, the statistical analysis considered the HN001 dose as categorical and numerical (up to quadratic order) variables. In addition, the average daily intake across the study and the IF consumed in the sampling day (last meal consumed) were considered as covariates. Both average daily and last-day IF intakes were included in the model as feed refusals were observed throughout the study, and they could have affected some of the analysed response variables.

The best polynomial model (linear or quadratic) for each response variable was selected after comparing higher- *v*. reduced-order models using the log-likelihood ratio test. This later test was also used to compare the best polynomial model with the model using dose as a categorical variable. For all response variables, the best fit model had dose as a numerical variable.

Some of the cytokines (IFN-*γ*, IL-4, IL-8 and TNF-*α*) were detected only in the plasma of a few piglets in each treatment (e.g. IL-8 was detected in only one piglet fed the formula with the low level of HN001). Therefore, a frequency analysis was conducted using a binary logistic regression using the Proc Glimmix procedure with 0 when the cytokine was not detected and 1 when the cytokine was detected.

The model diagnostics for each response variable were tested after combining the PROC UNIVARIATE and the ODS GRAPHICS procedures of SAS before comparing the means. A transformation of raw data was conducted when a response variable did not fulfil the model assumptions of normality and homoscedasticity. An outlier was removed when the Studentised residual was greater than 3. A predictor effect was considered significant when *P* < 0·05 and a trend when 0·051 < *P* < 0·10.

## Results

Dietary HN001 at either dose did not affect any of the growth performance parameters analysed (*P* > 0·05; Table 1). Jejunal tissue architectural features and tight junction gene expression as measured by RT–qPCR were also not influenced by HN001 in the IF (*P* > 0·05; Tables 2 and 3, respectively). In contrast, goblet cell numbers, crypt surface and claudin 1 gene expression in ileal tissues increased (*P* < 0·05) in piglets fed IF with HN001 at 1·3 × 10^5^ CFU/ml with the higher HN001 dose showing no additional effect. In addition, several architectural features of the ileum (goblet cells, Peyer’s patches and crypt surface) and expression levels of occludin in the jejunal tissue, as well as claudin 2 and tight junction protein 2 genes in the ileal tissue were influenced (*P* < 0·05) by both the average daily intake and the amount consumed in the last meal covariates.

**Table 1.** Growth parameters of piglets fed diets containing different levels of *Lactobacillus rhamnosus* HN001 (Standard errors of the mean)

	Probiotic (CFU/ml formula)*				
	0	1·3 × 10^5^	7·9 × 10^6^	sem	*P*
Initial body weight, kg	4·22	4·23	4·56	0·161	0·174
Final body weight, kg	9·32	9·33	9·92	0·296	0·191
Average daily intake, L	2·32	2·32	2·34	0·630	0·882
Average daily gain, g	243	243	255	8·6	0·344

CFU, colony formic units.

* Values are means and pooled standard error of the means, *n* 8 for each treatment.

**Table 2.** Architectural features of the jejunal and ileal tissues of piglets fed diets containing different levels of *Lactobacillus rhamnosus* HN001 (Standard errors of the mean)

	Probiotic (CFU/ml formula)*				*P*		
	0	1·3 × 10^5^	7·9 × 10^6^	sem	Probiotic	ADI	Meal†
Jejunum							
Goblet cells, #/villus	10·7	10·7	9·4	0·60	0·157	–	–
Villus length, µm	812	811	774	21·4	0·245	–	–
Villus surface, mm^2^	108	108	97·3	5·39	0·184	–	–
Crypt depth, µm	250	250	252	7·62	0·826	–	–
Crypt surface, mm^2^	14·2	14·2	14·1	0·72	0·888	–	–
VL/CD	3·31	3·31	3·18	0·14	0·574	–	–
Ileum							
Goblet cells, #/crypt	12·9	15·2	14·7	0·83	0·030Q‡,§	0·042	–
Peyer’s patches, mm^2^	1033	1028	1027	54·5	0·710	0·009	–
Crypt surface, mm^2^	18·8	25·7	22·4	1·58	0·012Q‖	<0·001	0·013

ADI, average daily intake across the whole study; CFU, colony formic units; VL/CD, villus length:crypt depth ratio.

* Values are means and pooled standard error of the means, *n* 8 for each treatment.

† Meal consumed on the sampling day.

‡ Q, quadratic effect for the probiotic level factor.

§ Globet cells, # = 4·86 + 1·9 × 10^5^ Dose – 2·32 × 10^12^ Dose^2^ + 0·003 ADI.

‖ Crypt surface, mm = –9·54 + 5·4 × 10^5^ Dose – 6·78 × 10^12^ Dose^2^ + 0·013 ADI – 0·001 meal.

**Table 3.** Normalised jejunal and ileal expression of genes related to intestinal permeability in piglets fed diets containing different levels of *Lactobacillus rhamnosus* HN001 measured by RT-qPCR (Standard errors of the mean)

	Probiotic (CFU/ml formula)*				*P*		
	0	1·3 × 10^5^	7·9 × 10^6^	sem	Probiotic	ADI	Meal†
Jejunum							
YWHAZ	22·5	22·5	22·4	0·20	0·717	–	–
Claudin 1‡	0·79	0·80	1·03	0·17	0·384	–	–
Claudin 2	1·21	1·21	1·09	0·18	0·645	–	–
Occludin	1·03	1·05	1·19	0·13	0·495	0·015	0·002
TJP1‡	1·05	1·05	0·89	0·15	0·376	–	–
TJP2	1·07	1·06	0·88	0·12	0·301	–	–
Ileum							
YWHAZ	22·2	22·2	22·0	0·19	0·752	–	–
Claudin 1	1·08	2·17	1·62	0·36	0·044Q§,‖	–	–
Claudin 2	1·03	1·09	1·00	0·21	0·581	–	0·007
Occludin	0·93	0·93	1·00	0·15	0·785	–	–
TJP1	1·00	1·00	0·93	0·08	0·553	–	–
TJP2	0·96	1·00	0·99	0·16	0·831	–	0·028

ADI, average daily intake across the whole study; CFU, colony formic units; YWHAZ, 14-3-3 protein zeta/delta (housekeeping gene); TJP, tight junction protein.

* Values are means and pooled standard error of the means, *n* 8 for each treatment. The values for YWHAZ are expressed as Ct values, while for the remaining genes as expressed as normalised values.

† Meal consumed on the sampling day.

‡ An outlier was removed from the piglets fed the diet without HN001.

§ Q, quadratic effect for the probiotic level factor.

‖ Claudin 1 = 1·08 + 8·54 × 10^6^ Dose – 1·07 × 10^12^ Dose^2^.

Transcriptome analysis by RNAseq showed that supplementation of IF with HN001 significantly altered gene expression (defined as |logFC| > 0·585 and false discovery rate < 0·05) in the jejunal tissue (Table 4) in a dose-dependent fashion. Compared with the control, low-dose HN001 significantly altered the expression of fifteen genes in jejunal tissues, whereas high-dose HN001 led to the differential expression of twenty-six genes. Of the fifteen jejunal genes altered by low-dose HN001, fourteen genes were (or tended to be) significantly changed in response to high-dose HN001.

**Table 4.** Differentially expressed genes of jejunal tissue transcriptomes from piglets fed diets containing different levels of *Lactobacillus rhamnosus* HN001

			Control *v*. Low		Control *v*. High	
Ensemble ID	Gene name	Description	logFC*	FDR†	logFC*	FDR†
ENSSSCG00000000875	NR1H4	Nuclear Receptor Subfamily 1 Group H Member 4			0·720	0·043
ENSSSCG00000001844	PLIN1	Perilipin 1	–3·852	0·018	–4·127	0·009
ENSSSCG00000001867	PSTPIP1	Proline-Serine-Threonine Phosphatase Interacting Protein 1	–0·940	0·042	–0·891	0·042
ENSSSCG00000003578	FGR	Tyrosine-Protein Kinase	–0·840	0·042	–0·843	0·026
ENSSSCG00000003756	LPAR3	Lysophosphatidic Acid Receptor 3	–0·941	0·042	*–0·823*	*0·075*
ENSSSCG00000003763	IFI44	Interferon Induced Protein 44	0·655	0·004	*0·433*	*0·087*
ENSSSCG00000004139	ADGRG6	Adhesion G Protein-Coupled Receptor G6			–0·928	0·020
ENSSSCG00000004961	ITGA11	Integrin Alpha-11			–0·660	0·036
ENSSSCG00000008157	IL18RAP	Interleukin 18 Receptor Accessory Protein	–0·969	0·018	–0·867	0·032
ENSSSCG00000008510	LTBP1	Latent Transforming Growth Factor Beta Binding Protein 1			–0·794	0·004
ENSSSCG00000008994	RBM46	RNA Binding Motif Protein 46			–1·274	0·028
ENSSSCG00000012141	CA5B	Carbonic Anhydrase 5B			–3·147	0·032
ENSSSCG00000012347	ALAS2	5'-Aminolevulinate Synthase 2			–2·077	0·020
ENSSSCG00000013067	PHEROC	Pheromaxein C subunit			–1·714	0·041
ENSSSCG00000013236	MYBPC3	Myosin Binding Protein C3			–1·761	0·044
ENSSSCG00000013490	PIP5K1C	Phosphatidylinositol-4-Phosphate 5-Kinase Type 1 Gamma			–0·610	0·009
ENSSSCG00000014149	MEF2C	Myocyte Enhancer Factor 2C	–0·937	0·008	–0·921	0·009
ENSSSCG00000014203	MCC	Colorectal Mutant Cancer Protein			–0·679	0·037
ENSSSCG00000014838	PGM2L1	Phosphoglucomutase 2 Like 1	–0·795	0·041	*–0·611*	*0·100*
ENSSSCG00000021917	HIPK4	Homeodomain Interacting Protein Kinase 4			–1·596	0·050
ENSSSCG00000024644	CIDEA	Cell Death Inducing DFFA Like Effector A	–3·716	0·042	–4·114	0·016
ENSSSCG00000025286	MCTP1	Multiple C2 And Transmembrane Domain Containing 1	–1·013	0·005	–0·859	0·020
ENSSSCG00000027266	PNPLA3	Patatin Like Phospholipase Domain Containing 3	–2·849	0·018	–2·509	0·032
ENSSSCG00000028282	SLC1A4	Solute Carrier Family 1 Member 4			0·988	0·032
ENSSSCG00000030626	ALDH1L1	Aldehyde Dehydrogenase 1 Family Member L1			–3·559	0·040
ENSSSCG00000033623	APOBR	Apolipoprotein B Receptor	–0·629	0·004	–0·605	0·007
ENSSSCG00000033822	THRSP	Thyroid Hormone-Inducible Hepatic Protein			–9·620	0·009
ENSSSCG00000037074		Uncharacterised			–1·233	0·032
ENSSSCG00000037358	SAA2	Serum Amyloid A-2	4·004	0·018	*0·111*	*0·971*
ENSSSCG00000040681	FABP4	Fatty Acid Binding Protein 4	–4·560	0·005	–4·660	0·006
ENSSSCG00000040719	KIAA0040	Uncharacterised	–0·629	0·042	*–0·537*	*0·085*

* logFC, log_2_ fold change. Positive LogFC value indicates higher expression in the HN001 group; negative LogFC indicates lower expression in the HN001 group. Italicised values indicate false discovery rate adjusted *P*-value > 0·05 (*n* 8 for each treatment).

† FDR, false discovery rate adjusted *P*-value from generalised linear model likelihood ratio test. Italicised values indicate FDR > 0·05. Genes with |logFC| > 0·585 (i.e. 1·5× fold change) and FDR < 0·05 considered significantly differentially expressed.

In most cases, regardless of HN001 dose, differentially expressed genes showed decreased expression after HN001 supplementation, with a few exceptions (e.g. Interferon Induced Protein 44). Genes that showed decreased expression levels (logFC > 0·583; false discovery rate < 0·05) in the jejunum after exposure to low-dose HN001 (compared with the control IF) included several genes associated with lipid metabolism (e.g. PLIN1, LPAR3, PNPLA3, APOBR, FABP4) and the immune response (e.g. IL18RAP). Many of these genes also showed reduced expression in response to high-dose HN001 (e.g. PLIN1, IL18RAP, APOBR) compared with the control IF. Genes that were down-regulated only by high-dose HN001 included those involved in immunity (ITGA11, LTBP1), cell migration, adhesion (ADGRG6, ITGA11) and the bile acid receptor (NR1H4). However, the gene expression levels in ileal tissues were not significantly altered by IF containing HN001 compared with the control IF (data not shown).

Plasma concentrations of IL-10 for the piglets fed the control IF were 3·1-fold lower (*P* < 0·05; Table 5) compared with those fed the high-dose HN001 IF, but similar to those fed the low-dose HN001 IF. The frequency of detectable plasma IL-8 tended to be lower (*P* = 0·09) for piglets fed the low-dose HN001 IF compared with those fed the control IF or the high-dose HN001 IF. Plasma concentrations of calprotectin increased (*P* < 0·05; Table 6) by 0·97 ng/ml per 10^6^ CFU HN001. The covariate average daily intake influenced plasma calprotectin. There was no effect (*P* > 0·05) of HN001 on the concentration of *α*1-antitrypsin inhibitor promoter in plasma and faeces, cortisol in plasma and faeces and calprotectin in faeces.

**Table 5.** Blood plasma cytokine concentrations of piglets fed diets containing different levels of *Lactobacillus rhamnosus* HN001 (Standard errors of the mean)

	Probiotic (CFU/ml formula)*							
	0		1·3 × 10^5^		7·9 × 10^6^		sem	*P*
IFN-*α*, ng/l plasma†	2·08		2·08		1·83		0·177	0·354
IFN-*γ*, frequency‡	0·50	0–57·4	0·13	0–26·4	0·63	0–81·9	0·157	0·113
IL-4, frequency‡	0·75	0–5·96	0·63	0–5·96	0·63	0–12·4	0·165	0·812
IL-8, frequency‡	0·63	0–93·0	0·13	0–19·0	0·75	0–37·7	0·149	0·090
IL-10, ng/l plasma	67·8		70·1		208		28·78	0·019L§,‖
IL-12 p40, ng/l plasma¶	2·89		2·89		2·80		0·176	0·725
TNF-*α*, frequency‡	0·50	0–210	0·13	0–126	0·63	0–260	0·157	0·113

CFU, colony formic units; IFN, interferon.

* Values are means and pooled standard error of the means, *n* 8 for each treatment.

† A reciprocal transformation of the raw data was required to achieve the model assumptions of normality and homoscedasticity.

‡ The frequency analysis was conducted using a binary logistic regression with 0 when the cytokine was not detected and 1 when it was detected. The reported values represent the ratio of piglets with cytokine concentrations detected. The values in brackets represent the range of cytokine concentrations (ng/l plasma).

§ L, Q, linear or quadratic effect for the probiotic level factor, respectively.

‖ IL-10, ng/l plasma = 67·8 + + 1·8 × 10^5^ Dose.

¶ A natural log transformation of the raw data was required to achieve the model assumptions of normality and homoscedasticity.

**Table 6.** Plasma and faecal concentrations (ng/ml) of *α*1-antitrypsin inhibitor promoter, calprotectin and cortisol of piglets fed diets containing different levels of *Lactobacillus rhamnosus* HN001 (Standard errors of the mean)

	Probiotic (CFU/ml formula)*				*P*	
	0	1·3 × 10^5^	7·9 × 10^6^	sem	Probiotic	ADI
Plasma						
A1T1	278	279	295	28·5	0·702	–
Calprotectin	36·8	36·5	44·5	3·63	0·034L†,‡	0·002
Cortisol	76·3	76·7	99·1	9·42	0·123	–
Faeces						
A1T1	12·8	12·8	14·2	1·74	0·584	–
Calprotectin	98·5	98·1	73·4	13·6	0·177	–
Cortisol	114	114	124	3·4	0·060	–

A1T1, *α*1-antitrypsin inhibitor promoter; ADI, average daily intake across the study; CFU, colony formic units.

* Values are means and pooled sem, *n* 8 for each treatment.

† L, linear effect for the probiotic level factor.

‡ Plasma calprotectin, ng/ml = –27·4 + 1·29 × 10^6^ Dose + 0·027 ADI.

## Discussion

Feeding IF containing HN001 to piglets starting at 10 d of age for 24 d influenced architectural features of the ileal tissues and gene expression levels in jejunal tissues but did not impact on growth parameters.

The RNAseq results provided evidence that HN001 impacts the jejunal tissue transcriptome in a dose-dependent manner. The mucus layer of the small intestine is thin, which increases the opportunities for probiotic bacteria to interact directly with the epithelial cells^(24)^.

**Fig. 1.**
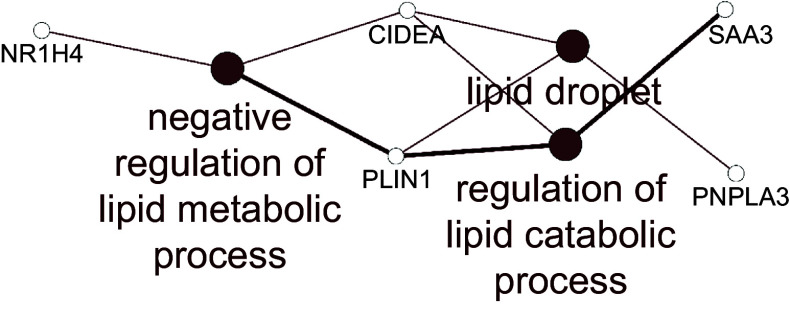
Network of Gene Ontology Biological Processes and KEGG pathways enriched among differentially expressed genes arising from jejunal tissue of piglets fed both low and high dose of *Lacticaseibacillus rhamnosus* HN001. Filled circles indicate processes/pathways, and empty circles genes. Lines link pathways that share common genes and genes mapped to a particular pathway. KEGG, Kyoto Encyclopedia of Genes and Genomes.

For the jejunum, there were insufficient numbers of altered genes at each HN001 dose to conduct enrichment analysis of biological processes. Therefore, the lists of differentially expressed genes for each HN001 dose were combined and analysed for enrichment of Gene Ontology Biological Processes and Kyoto Encyclopedia of Genes and Genomes pathways using the ClueGO app^(19)^ for Cytoscape. The combined enrichment analysis showed that differentially expressed genes were involved in several aspects of lipid metabolism (Fig. 1). For example, the mRNA abundance of the gene *angptl4*, which is involved in lipid metabolism, was higher in the small intestine of infant mice given HN001 in drinking water^(12)^.

**Fig. 2.**
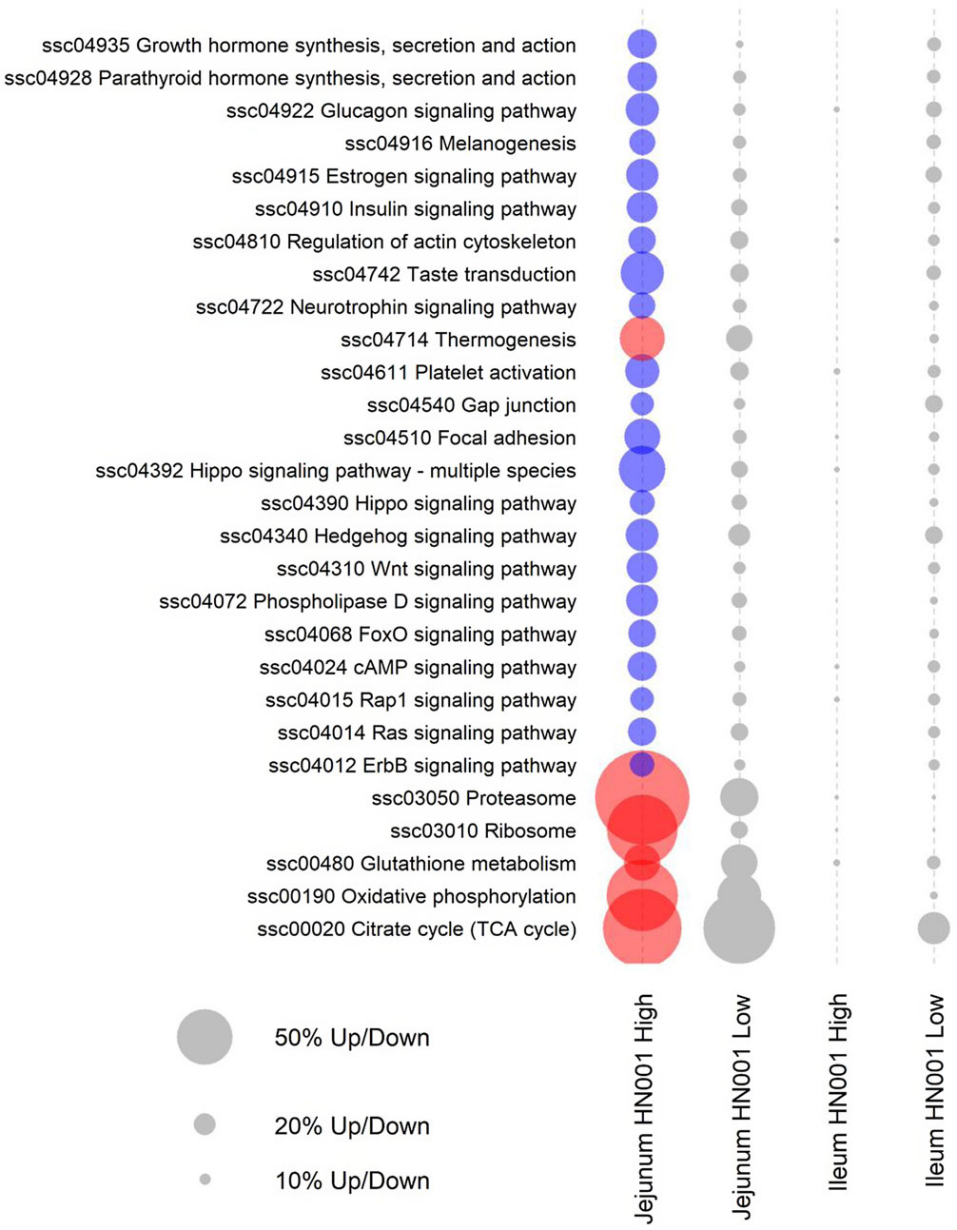
KEGG pathways differentially expressed by GSEA (FDR < 0·05) in jejunal and ileal tissues of piglets fed diets containing different levels of *Lacticaseibacillus rhamnosus* HN001. Red or blue circles indicate significantly higher or lower expression compared with the control group, respectively. Grey circles indicate pathways not differentially expressed (FDR > 0·05) (*n* 8 for each treatment). The size of a circle is proportional to the number of genes up or down-regulated. KEGG, Kyoto Encyclopedia of Genes and Genomes; GSEA, gene set enrichment analysis; FDR, false discovery rate.

Further pathway analyses were carried out on the jejunal transcriptome data using GSEA with gene annotated to individual Kyoto Encyclopedia of Genes and Genomes pathways assigned as gene sets. GSEA analysis also highlighted differences in the jejunal transcriptome of piglets fed high-dose HN001 IF (Fig. 2). Even though the low-dose HN001 IF did not affect pathway gene expression, the overall pattern closely matched that observed with the high-dose HN001 piglets, supporting that changes induced by HN001 were dose-dependent. Similar to jejunal individual gene data, the most affected genes in pathways showed decreased expression levels. These included pathways involved in glucose metabolism (glucagon signalling and insulin signalling pathways), hormone metabolism, neurotrophin signalling, cell structure (Rap1 signalling, ErbB signalling, hippo signalling, regulation of actin cytoskeleton, focal adhesion, gap junction) and signalling pathways that regulate cell cycle and apoptosis, such as FoxO, cAMP and Ras signalling pathways. The few pathways with increased expression were involved in energy metabolism (e.g. thermogenesis, oxidative phosphorylation, TCA cycle), protein cycling (e.g. proteasome and ribosome pathways) and glutathione metabolism, which has important roles in defence against reactive oxygen species and detoxification of xenobiotic compounds. The expression levels of genes related to permeability were not affected by the intake of HN001 IF at either dose.

In contrast to the jejunum, ileal histology (crypt surface and the number of goblet cells) increased when piglets received HN001 at either dose. This finding could be ascribed to the relatively high abundance of HN001 as reported in adult mice (e.g. 30 *v*. 3 % of total 16S rRNA for ileal and jejunal digesta, respectively)^(12)^. As small intestinal crypts have an array of epithelial cells (e.g. enterocytes, goblet cells, Paneth cells), an increase in ileal crypt surface areas in HN001-fed piglets suggests an increase in epithelial cell numbers, which aligns with the increased goblet cell numbers observed for these piglets. Goblet cells produce mucin, and the number of goblets cells is related to the thickness of the mucus layer^(25)^. Thus, it is possible that piglets fed HN001 had thicker ileal mucus layers than those fed the control IF. Furthermore, adult mice fed HN001 also showed an increase in the rate of duodenal enterocyte migration, and this was interpreted as a potential mechanism to reduce pathogen adherence to the epithelium^(12)^. However, this was not measured in the present study.

Despite the effect of HN001 supplementation on the ileal histology, HN001 did not affect the ileal tissue transcriptome. This finding was somewhat surprising as the Peyer’s patches play an important role in bacteria–host interactions^(24)^, and based on results from adult mice^(12)^, a high abundance of HN001 reached the ileum. Claudin 1 was not detected in the RNAseq results, but the qPCR results showed that Claudin 1 was the only tight junction protein gene analysed here that had increased expression levels in the ileal tissue with the intake of both doses of HN001. Previous studies have shown that HN001 increased the transepithelial electrical resistance (a measurement of the integrity and permeability) in Caco2 cells^(26)^ and Caco2:HT29-MTX epithelial co-culture cells^(27)^ and increased the expression levels of tight junction proteins (tight junction protein 2 and occludin)^(27)^. Based on this *in vitro* evidence, it could be expected that HN001 would improve the ileal barrier integrity of piglets, as shown elsewhere in claudin-1-deficient mice^(28)^. Therefore, it is possible that gene expression over the whole ileum may have diluted specific effects on specific ileal tissue components, such as the epithelial cells or Peyer’s patches.

Blood plasma concentrations of the anti-inflammatory cytokine IL-10 increased as the level of HN001 increased in IF (17·7 ng/l per 10^6^ CFU). However, low-dose HN001 tended to reduce the frequency of detectable pro-inflammatory IL8. These findings might suggest that HN001 may have beneficial effects on health by increasing the production of anti-inflammatory cytokines while reducing (or not affecting) pro-inflammatory cytokines. In healthy mice, it has been shown that the intake of HN001 enhanced the immune response^(9)^, and this enhancement is dose-dependent, with better responses with higher doses^(29)^.

To determine the dose effect of HN001 on GIT health, markers of inflammation (calprotectin), stress (cortisol) and intestinal protein loss (*α*1-anti trypsin) were measured in faecal and blood samples. Blood and faecal cortisol and *α*1-anti trypsin were not affected by the HN001 dose. Blood, but not faecal, concentrations of calprotectin were higher for the piglets fed high-dose HN001. Increased calprotectin in both faeces^(30,31)^ and blood^(32)^ has been related to chronic and acute inflammation. For instance, the calprotectin concentration in blood plasma was 4-fold higher in patients with complicated acute appendicitis compared with the control^(32)^. However, in the present study, the faecal concentration of calprotectin was not modulated by the high dose of HN001. When considered the mean plasma blood concentrations of IFN gamma and TNF-*α* in the present study, piglets fed the high dose of HN001 had 29 % and 8 % more of IFN-*γ* and TNF-*α*, respectively, than those fed the control IF. An *in vitro* study has shown that IFN-*γ* and TNF-*α* increased the expression of calprotectin (S100A8) in macrophages^(33)^. Thus, the higher concentration of IFN-*γ* and TNF-*α* in the piglets fed the high dose of HN001 could partially explain the raised level of blood plasma calprotectin. It is also possible that the increased blood calprotectin levels may be related to increased NK cell and phagocyte activity observed in adult human after HN001 intake for 3 weeks^(34)^.

Some response variables analysed were influenced by either (or both) the average daily intake during the whole experimental period or (and) the amount of milk consumed in the last experimental day. However, for some variables (e.g. number of ileal goblets cells), the use of these covariates helped reduce the model’s error and detect differences across treatments. This result highlights the importance of controlling and recording food intake in piglet studies and including it as a covariate in the statistical analysis.

In conclusion, in a piglet model of the human infant, intake of HN001 had important effects on the small intestine in terms of both architecture (e.g. increased number of goblet cells) and gene expression, as well as systemic changes in terms of blood plasma cytokine concentrations (e.g. IL-10). Furthermore, these effects were mostly achieved at a lower dose of HN001 typical of commercial IF. Together, these results suggest that supplementation of IF with HN001 might modulate the small intestinal tissue maturation in the piglet model.
